# Measurements and Visibility of the Pancreatic Ducts on Computed Tomography in 78 Cats Without Clinical Evidence of Pancreatitis

**DOI:** 10.3390/ani15192857

**Published:** 2025-09-30

**Authors:** Abby Caine, Man-Hei Ma, Mike Herrtage, Tim Sparks, Marie Aude Genain

**Affiliations:** 1 Department of Veterinary Medicine, University of Cambridge, Madingley Road, Cambridge CB3 0ES, UK; 2 Dick White Referrals, Linnaeus Group, Station Farm, London Road, Six Mile Bottom, Cambridgeshire CB8 0UH, UK; 3 Waltham Petcare Science Institute, Freeby Lane, Waltham on the Wolds, Melton Mowbray, Leicestershire LE14 4RT, UK; tim.sparks@effem.com

**Keywords:** computed tomography, feline, pancreatic duct diameter, duodenal papilla, domestic cats

## Abstract

Imaging is commonly used to help diagnose pancreatic disease in cats. In order to establish normal computed tomography (CT) measurements for the pancreatic ducts in cats, images of 78 cats without clinical evidence of pancreatic disease, and with normal DGGR (1,2-o-dilauryl-rac-glycero-3-glutaric acid-(6′-methylresorufin) ester lipase values, were evaluated retrospectively by two reviewers. The left pancreatic duct measured 1.4 ± 0.8 mm (mean ± standard deviation), the right pancreatic duct measured 1.1 ± 0.5 mm, and the common duct measured 1.6 ± 0.8 mm. All ducts were better visualised post-contrast, with the left pancreatic duct identified most frequently (not visualised on post-contrast images in only 3% of cases). There was visibility of the right and common hepatic ducts less frequently, not observed post-contrast in 22 and 20% of cases, respectively. The duodenal papilla measured 2.8 ± 0.7 mm in diameter. It had an HU of 43 ± 14 pre-contrast and 109 ± 32 post-contrast. It was identified in all but 6% of cases on post-contrast images. This study shows that the pancreatic ducts and duodenal papilla can be seen on post-contrast images and provides normal ranges of size for the pancreatic duct and duodenal papilla.

## Simple Summary

Cats often suffer from diseases of their pancreas, which is an abdominal organ that produces enzymes that help digest food and also produces hormones, including those that regulate blood sugar. Vets use a type of imaging using X-rays called computed tomography (CT) to look at internal organs to help diagnose for pancreatic disease. It is important to know what internal organs normally look like so abnormalities can be recognised. This study looks at the CT appearance of the pancreas in a large group of 78 healthy cats and provides normal sizes for the internal ducts in the pancreas. The average size is 1.4 mm (left duct), 1.1 mm (right duct), and 1.6 mm where the two ducts join (common duct). Many CT studies of the abdomen in cats will include extra images acquired after a contrast agent which contains iodine is injected into the vein. This contrast agent highlights blood vessels and internal organs with a high blood supply, and this study shows that vets need the images after contrast to be able to reliably see certain small structures, including the duodenal papilla (a small round structure that marks where the pancreatic duct enters the bowel).

## 1. Introduction

Pancreatic disease is recognised as a common and potentially significant pathology in cats, with postmortem studies identifying evidence of pancreatitis in 67% of cases [1]. Pancreatitis is inflammation of the exocrine pancreatic tissue. It is categorized into two presentations: acute and chronic forms. The acute form has the histopathological evidence of interstitial oedema and necrosis in the mesenteric fat and neutrophilic inflammation in the pancreatic parenchyma, while the chronic form shows lymphocytic inflammation, fibrosis and cystic acinar degeneration [1,2,3]. Studies have identified a greater prevalence of chronic pancreatitis than acute pancreatitis [1,4] with some cases showing both concurrently, and with authors suggesting that the two forms could reflect a continuum of disease in some patients [2]. Given the distinction is based on histopathology, it may not be feasible to distinguish in clinical patients without biopsy, and some studies suggest it may not be distinguished clinically [5].

The aetiopathogenesis of pancreatitis is debated [2,3] and may be varied, with some specific causes identified, including trauma (such as a road traffic accident or high-rise injury from a fall) [6], surgery (potentially, hypotension during anaesthesia is more significant than the surgical manipulation of the pancreas), and some infectious agents, including parasites such as toxoplasma [7], as well as other causes, including a number of viruses [8]. Neoplasia of the pancreas can trigger pancreatitis, however pancreatic neoplasia is relatively uncommon in cats [9,10]. Most cases of pancreatitis are considered idiopathic [2,3].

Pancreatitis may be identified on its own; however, it can be seen concurrently with other diseases, including inflammatory bowel disease, and with cholangiohepatitis. If all three inflammatory processes are present in one patient, this has been termed triaditis [2,11], although the association of other diseases with feline pancreatitis remains debated. The pathogenesis of this association between diseases is not clear. There is speculation over whether the association could be anatomic, since the common bile duct and pancreatic duct join at the ampulla of Vater immediately before entering the major duodenal papilla [10]. Alternatively, it could be speculated that there is systemic pathology, such as immune-mediated disease, that concurrently affects multiple organs [2] to explain the observed co-morbidity.

Diagnosis may be challenging ante mortem, given relatively non-specific clinical signs [4,12]. It is based on a combination of history, clinical presentation, bloodwork and diagnostic imaging. Routine bloodwork shows a variety of abnormalities, including anaemia, elevated ALT (alanine aminotransferase) and ALKP (alkaline phosphatase), and hypoglycaemia [5]. More specific tests to assess pancreatic function are available, including DGGR (1,2-o-dilauryl-rac-glycero-3-glutaric acid-(6′-methylresorufin)), ester lipase [13], and fPLI (serum feline trypsin-like immunoreactivity) [14]. When compared, DGGR lipase and fPLI show substantial agreement between the two assays; however, there was only fair agreement between fPLI and ultrasound diagnosis [15]. fPLI was judged to be sensitive for cases of moderate to severe pancreatitis [16] and is recommended to be used as part of the diagnostic tests to identify pancreatitis. However, it may miss subtle cases [2,3].

Due to the complex interaction with pathology in other organs and the difficulty in confirming a diagnosis of pancreatitis with pancreatic function tests alone, diagnostic imaging plays an essential role in the diagnosis of pancreatitis. Ultrasound is the most common imaging modality involved [17]. Studies report variable sensitivity when ultrasound is compared to biopsy, from 24% [18] to 80% for severe pancreatitis in a separate study [16]. Ultrasonographic features associated with pancreatitis diagnosed with fPLI include hyperechoic peripancreatic fat, which was an ultrasound feature associated with pancreatitis in two studies [19,20]; with other features identified, including irregular margins [19]; hypoechoic appearance of the pancreas [20]; increased thickness of the left limb [19]; or increased size [20]. In some cats, pancreatic duct dilation has been associated with pancreatitis [21]. A mean ± standard deviation (SD) of 0.8 ± 0.25 mm (range 0.5–1.3 mm) has been reported for pancreatic duct size in cats [22]. However, a weak linear correlation between increasing age and increasing duct size has also been recognised in cats [23]. A study specifically evaluating the pancreatic duct size in older cats showed that the pancreatic duct in 15 healthy cats of over 10 years of age (mean 13 years) was 1.3 ± 0.4 mm (0.6–2.4 mm) at the widest point [24]. In the same paper, a retrospective study of 1445 cats [24] also showed a correlation between pancreatic duct width and age, yet no correlation of duct width with pancreatic disease. Given that pancreatic duct dilation was identified more frequently in older cats, it is difficult to justify pancreatic duct dilation as a criterion to diagnose pancreatitis on ultrasound in the absence of other findings.

The duodenal papilla has been identified in cats on ultrasound, with reference ranges of 2.9–5.5 mm width [22] and is shown to have a slight difference in size between transverse and longitudinal orientations [25].

Computed tomography (CT), as an imaging modality, is often used to diagnose pancreatitis in humans and can avoid some of the problems arising from acute paralytic ileus in the early phase of acute pancreatitis that can hamper ultrasound imaging [26]. CT of the pancreas has been described in veterinary medicine [27]. Studies have evaluated the use of CT in the diagnosis of feline pancreatitis [16], with estimates showing it was similar to the relatively poor performance of ultrasound (20% sensitivity) [18]. However, the specific evaluation of the pancreatic ducts on CT has only been assessed in a low number (16) of normal cats, with that study suggesting that there may be some difference in the appearance of the ducts in cats which were positive for fPLI [28]. Therefore, analysis of the pancreatic ducts may play a more significant role in identifying cats with pancreatitis on CT in comparison with ultrasound.

This current study aims to describe the CT appearance of the pancreatic ducts and duodenal papilla in a larger cohort of cats with no clinical or biochemical evidence of pancreatic disease. Based on the previous literature of CT appearance in 16 cats, it is hypothesised that the majority of cats will have some or all of the pancreatic ducts visualised on CT. However, there may be a difference in visibility between segments of the pancreatic duct. It is also hypothesised that there will be an improvement in the ability to identify all parts of the pancreatic ducts and duodenal papilla following contrast administration.

## 2. Materials and Methods

This is a retrospective study to evaluate the appearance of the pancreatic ducts on CT in cats with no clinical or biochemical evidence of pancreatic disease.

One hundred of the most recent CTs of the thorax, abdomen or both in cats, acquired from institution A (The Queen’s Veterinary School Hospital, QVSH) and institution B (Dick White Referrals, DWR), were collected for a separate study evaluating the pre- and post-contrast CT appearance of the hepatobiliary tract in cats without evidence of hepatobiliary disease. The indication for CT was variable; however, no cats that had a clinical suspicion or final diagnosis of hepatobiliary or pancreatic disease were included. Appendix A includes the final diagnosis of the patients included in the study.

Institution A acquired images with a Toshiba Aquillon (16 slices) CT (Toshiba, Minato, Tokyo, Japan); institution B acquired images with a GE Revolution Evo (64 slices) CT (GE healthcare, Cincinnati, OH, USA) after July 2023 (9 cases) and PNMS MX (16 slice), (Phillips Neusoft Medical systems, Shenyang, China) prior to July 2023 (34 cases).

As per protocols at both institutions, cats had food withheld prior to the procedure, were under general anaesthesia administered based on the attending anaesthetist’s preference and were placed in sternal recumbency for the procedure. Images were acquired prior to and post administration of iodinated intravenous contrast media (2 mL/kg of Iohexol 300 mgI/mL (Omnipaque, GE Healthcare, Chicago, IL, USA)).

Specific imaging parameters for CT acquisition for each machine are recorded in Appendix B.

Patients were included if:

- There was contemporaneous standard bloodwork that included normal DGGR lipase, ALT (alanine transferase) or ALKP (alkaline phosphatase).
- There was no clinical evidence of pancreatic disease nor indication in the history of previous pancreatic disease.
- There was no final diagnosis of pancreatic disease identified in the records.

Patients were excluded if:

- An ultrasound was performed near the time of CT, and pancreatic abnormalities were identified
- fPLi was performed and was abnormal.
- Images were of inadequate quality or did not include the entire pancreas and pancreatic ducts for evaluation.

Images were reviewed by two authors (one ECVDI resident, one ECVDI diplomate) independently with a subset for intraclass correlation read at two time points with 6 weeks between review sessions. Both pre- and post-contrast series were available at the time of review, with the ability to reconstruct the images using 3D multiplanar reconstruction (MPR) as necessary using a DICOM viewer (Osirix, version 14.0.1; Pixmeo SARL, 266 Rue de Bernex—CH1233 Bernex—Switzerland).

### 2.1. The Following Measurements Were Taken

#### 2.1.1. Measurements and Visibility of the Pancreatic Ducts

**Figure 1:**
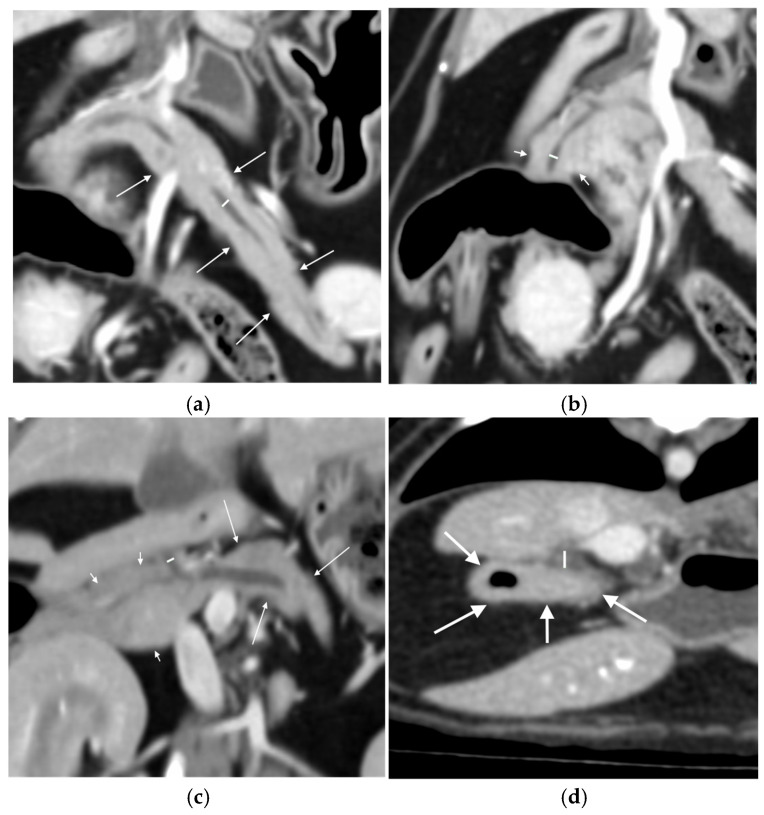
Locations of measurements of the left, right and common pancreatic ducts and the duodenal papilla: (**a**) measurement of the width (white line) of the left pancreatic duct at its widest point, seen on the dorsal plane multiplanar reconstruction in the left lobe of the pancreas (white arrows); (**b**) measurement of the width (white line) of the right pancreatic duct at its widest point, seen on the dorsal plane multiplanar reconstruction in part of the right lobe of the pancreas (white short arrows); (**c**) measurement of the width (white line) of the common pancreatic duct, seen on the dorsal plane multiplanar reconstruction as it exits the pancreas between the right lobe of the pancreas (white short arrows) and the left lobe of the pancreas (white longer arrows); and (**d**) diameter of the duodenal papilla (white line) measured on the post-contrast transverse image at the level where it enters the duodenum (white arrows).

- Width from outer layer to outer layer, and Likert visibility scale [29] pre- and post-contrast, of the left pancreatic duct at its widest point on a dorsal plane reconstruction (Figure 1a).
- Width from outer layer to outer layer, and Likert visibility scale pre and post-contrast, of the right pancreatic duct at its widest point on a dorsal plane reconstruction (Figure 1b).
- Width from outer layer to outer layer, and Likert visibility scale pre and post-contrast, of the common pancreatic duct as it exits the pancreatic parenchyma on a dorsal plane reconstruction (Figure 1c).
- Evaluate each segment for any subjective thickening or irregularity of the walls and for the presence of any mineral attenuation within the duct (subjectively assessed as being distinctly visible on bone window, WL 300, WW 1500).

#### 2.1.2. Measurements of the Duodenal Papilla

**Figure 2:**
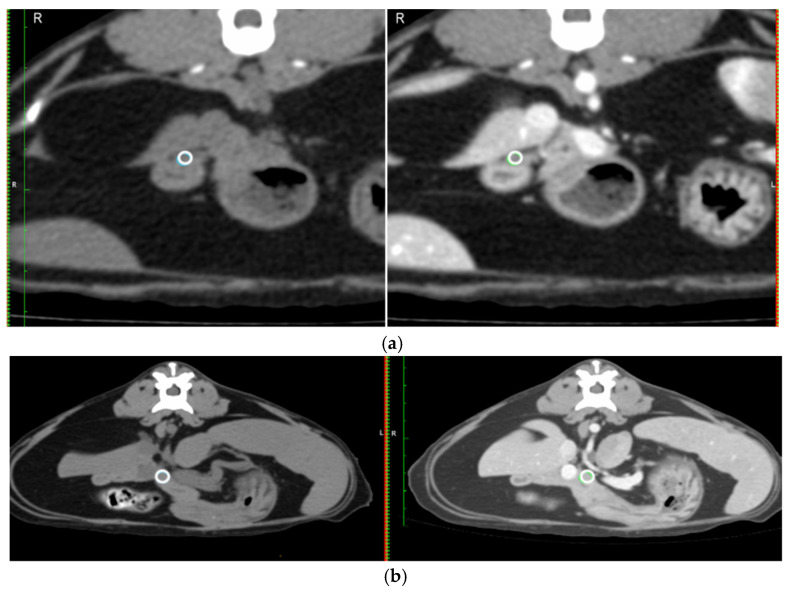
Placement of a ROI to measure the HU in the duodenal papilla and thepancreas: (**a**) a ROI placed on the duodenal papilla on the transverse post-contrast images (white circle on the right hand image) and copied across to the same place pre-contrast (white circle on the left hand image); and (**b**) ROI placed on the body of the pancreas on the transverse post-contrast images (white circle on right hand image), avoiding the pancreatic duct and copied across to the same place pre-contrast (white circle on left hand image).

- Mean Hounsfield unit (HU) on a circular region of interest (ROI) placed over the duodenal papilla pre- and post-contrast on the transverse image (Figure 2a).
- Likert visibility scale of the duodenal papilla pre- and post-contrast.
- Diameter of the duodenal papilla post-contrast taken perpendicular to the duodenal wall on the transverse image (Figure 1d).

#### 2.1.3. Pancreatic Parenchyma

- The width of the pancreas was measured at 90° to the long axis of the pancreas in the left and right limb at the widest point of the respective limb.
- The mean HU for the same circular ROI, pre- and post-contrast in a section of the pancreas at the level of the pancreatic body (Figure 2b).

All measurements were taken in mm rounded to 0.1 mm. HU were recorded in whole numbers.

Visibility was recorded using a 5-point Likert scale [29], defined as:

5 = entire segment/structure visible

4 = most/more than half of the segment/structure visible

3 = segment/structure partially visible

2 = segment/structure hardly visible but can be identified

1 = segment/structure not visible

### 2.2. Statistical Analysis

Data are summarised as mean with standard deviation and range, for each reviewer separately, and also as a combined sample.

Agreement within and between reviewers was evaluated using intraclass correlation (ICC) for the continuous and ordinal data. Intra-rater agreement was performed separately for reviewer 1 and 2 based on a subsample of 20 images assessed 6 weeks apart using two-way, random effects, absolute agreement ICC (2,1) calculated in R4.3.3. Interclass agreement between reviewers was calculated in the same way but based on all images meeting the inclusion criteria.

Comparisons between pairs of values used Wilcoxon signed-rank tests to assess the difference in visibility between pre- and post-contrast images, separately for each reviewer. Cat age was divided into two groups (1–9 years, 10+ years) and pancreatic duct size measurements were compared between these two groups using Mann–Whitney tests adjusted for ties, separately for each reviewer. Significance was taken as *p* < 0.05. Analysis was undertaken in R4.3.3 and Minitab21.

## 3. Results

Of the 100 initial cases, two were excluded as the entire pancreas was not included, and a further 20 were excluded due to mild elevations in DGGR lipase. This yielded 78 cases that fulfilled all inclusion criteria, i.e., 35 from institution A and 43 from institution B.

In the study population, breeds included eight Maine Coon, three British Short Hair, two Ragdoll, two Domestic Long Hair, two Siamese, and one each of Burmese, Egyptian Mau, Oriental Short Hair, Persian and Sphynx; the remainder were Domestic Short Hair cats (56).

The mean age was 9.2 years, ranging from 1 to 14 years. 32 cases were aged 1–9, 46 cases were aged 10–14. There were 38 neutered female cats, and the remaining 40 were neutered male cats.

### 3.1. Measurements Obtained from Imaging

#### 3.1.1. Pancreatic Ducts

Measurements of the diameter of the pancreatic ducts at different levels are presented in Table 1. Table 2 summarises the data for two age groups of cats, aged 1–9 and 10 years and over, respectively. Measurements did not differ significantly between the two age groups.

**Figure 3:**
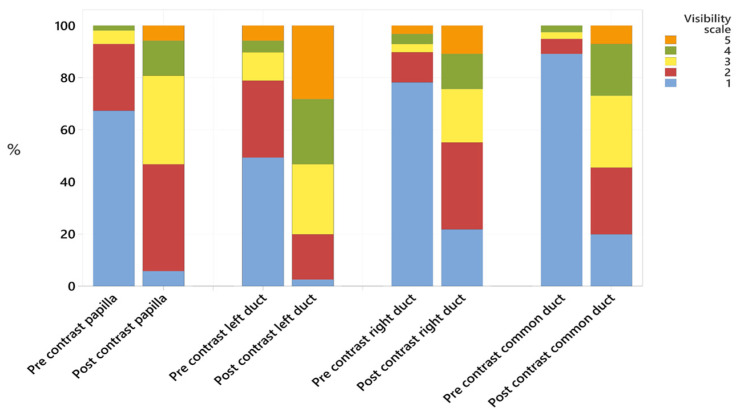
Stacked bar chart showing the percentage of cases to which each Likert scale was applied, comparing pre- and post-contrast visibility for the duodenal papilla, the left, right and common pancreatic ducts.

Combining data from both reviewers, the left, right and common pancreatic ducts were, respectively, graded as not visible (Likert grade 1) pre-contrast in 49%, 78% and 89% of the cases; and in 3%, 22%, and 20% of the cases post-contrast. The Likert score recorded for each segment of the pancreatic ducts is presented in Table 1. All structures had a significantly higher visibility score post-contrast (all *p* < 0.001, Figure 3, Table 1). No cases had any mineral attenuation noted within the pancreatic ducts. All pancreatic ducts were defined as smoothly marginated.

#### 3.1.2. Duodenal Papilla Appearance

The duodenal papilla diameter measured 2.8 ± 0.7 mm (mean ± standard deviation), with a range of measurements of 1.3–5.3 mm. The duodenal papilla could not be identified (Likert grade 1) in 68% of cases prior to contrast, but in only 6% of cases following contrast administration. The mean HU of the ROI of the duodenal papilla pre contrast was 43 ± 14 (range: 1–79). Post-contrast, this increased to 109 ± 32 (range: 47–197).

#### 3.1.3. Pancreatic Parenchyma

The mean ± SD (range) width of the left limb was 6.9 ± 1.4 mm (3.8–9.7), and for the right limb, it was 5.8 ± 1.2 mm (2–8.3). The HU for the pancreas, pre-contrast, was 56 ± 13 (range: 33–89). This increased to 129 ± 27 (range: 77–191) after contrast. The pancreatic parenchyma was judged to be homogenous both pre and post-contrast.

### 3.2. Statistical Analysis

Intraclass correlation coefficient (ICC) was similar for the left and right pancreatic duct width (0.73 and 0.76, respectively), with values 0.75–0.9 classified as good [30]. The ICC was lower for the common duct and the duodenal papilla, at 0.22 and 0.23, respectively (agreement classified as poor [31]); see Table 3.

Interobserver agreement is presented in Table 3. There were differences in consistency of assessing visibility between readers, and interobserver assessment of visibility was poor to fair [31]. In all but one case the intra- and inter-agreement on visibility was higher post-contrast than pre-contrast.

## 4. Discussion

CT measurements of the pancreatic ducts in a large cohort of cats were provided. In this study, the mean width of the left pancreatic duct was larger than the right, with the mean ± SD width for the left pancreatic duct given as 1.4 ± 0.8 mm (range: 0.6–7.6 mm). This is slightly larger than the mean and range given for the pancreatic duct previously published for ultrasound (measured in the left pancreatic limb at 0.8 ± 0.25 mm (range 0.5–1.3)) [22].

In a previous study, measuring duct sizes in 16 normal cats on CT [28], the mean and range for the left pancreatic duct were reported as 1.7 mm (range: 0.9–2.9), slightly higher than the current study although with a smaller range. The mean measurement of the right pancreatic duct was 1.5 mm (range: 1.1–2.8) in the previous CT study [28]. This measurement, again, is slightly higher compared to the mean and range found in the current study, 1.2 ± 0.5 mm (range: 0.5–3.2). The previous CT study identified that increased duct size was associated with pancreatitis independently of age; however, there were only low numbers to compare different ages of cats, and the study was unable to age-match the normal group with the pancreatitis group. In the current study, for cats aged 10+ years, the mean ± SD left pancreatic duct size was 1.5 ± 0.9 mm (range: 0.6–7.6), with the mean ± SD and range given on ultrasound for this age group resulting very similar in the previous study, at 1.3 ± 0.4 mm (range: 0.6–2.4) [24]. No significant difference was detected in the duct size of any segment between the cats in the two age groups in the current CT study.

Regarding the appearance of the duct, in the current study, there were no pancreatoliths identified in this population of cats which were without clinical evidence of pancreatitis, which is consistent with the findings in the group of normal cats in the previous CT study [28]. That same study indicated that cats with pancreatitis were more likely to demonstrate an irregular or beaded appearance of the pancreatic duct on CT, though normal cats had a smoothly marginated duct. And similarly, an irregular appearance was not seen in any cat in this current study of cats without evidence of pancreatitis.

The left pancreatic duct was identified in 16/16 normal cats in the previous study on CT, similarly to the current study, where it was identified in nearly all cases (graded as not visible in 3% of cases post-contrast). The right pancreatic duct was not visualised in 3/16 (19%) patients in the previous study, which is consistent with the current study, where it was not visualised in 22% of cases post-contrast. The identification of the common pancreatic duct as it exits the pancreas was not described in the previous study on the CT of cat pancreatic ducts; however, it was identified in 80% of CT examinations in this cohort of cats without clinical evidence of pancreatitis and was measured at 1.6 ± 0.8 mm (range: 0.6–4.6).

The duodenal papilla was identified in 94% of patients in post-contrast images. This study reports the measurement of the feline duodenal papilla given on CT, and the mean diameter of 2.8 ± 0.7 mm (range: 1.3–5.3) obtained on transverse images is consistent with the range of measurements reported in previous ultrasound studies. The method of measurement of the diameter obtained in our study corresponds most closely to the height of the papilla on the transverse plane obtained in a study that measured the papilla in three planes with ultrasound, in which the mean height was given as 2.47 ± 0.63 mm [22,25]. The appearance of a small circular contrast enhancing structure seen on CT is similar to that described for the CT appearance of human duodenal papillae [32]. It should be noted that papilla visibility prior to contrast enhancement was very poor.

When comparing with the mean and range given for pancreatic thickness in previous studies of the ultrasound appearance of the pancreas in normal cats, where the left limb mean is reported as 5.6 mm (95% reference intervals 2.6–9.5 mm) [23] in one paper, and in a different paper, the mean is reported as 5.4 mm (range 3.4–9 mm) [22]; the thickness of the left limb of the pancreas in this current study was within the range, but the mean was found to be slightly higher than previously published (6.9 mm). As the range in this current study is similar to previously published values, this supports the suggestion that these patients did not have pancreatic disease.

The thickness measured in the right limb in this current study (5.8 mm, range 2–8.3 mm) exceeds that provided in a single previous publication, i.e., of a mean of 4.5 mm (range 2.8–5.9 mm) [22]. The previous ultrasound study is, however, based on only 19 cats, and the other study referenced [23] for the left limb did not offer a range for the right limb, since it was not usually seen well enough with ultrasound to take measurements. However, in this current CT study, the right limb could be visualised in all cats well enough to take measurements, which could explain why the mean and range were larger for this limb in this modality.

The HU for the pancreas, with a mean of 55 pre-contrast and 129 post-contrast, is similar to a previous paper documenting the HU of the pancreas in healthy cats measured on triple phase contrast enhanced CT [33].

As might be expected, there was a difference in reviewer confidence in visualising each structure, which, to some extent, may be based on experience. One reviewer (reviewer 2) also provided slightly higher Likert scores on the second read which could indicate a learning curve leading to a greater confidence with time. Generally, structures that were better visualised (higher Likert score) had better performance on inter and intra-observer agreement, as may be expected.

### Limitations

The use of multiple CT scanners in the acquisition of the images for these cases can hamper the comparison of structures, and, in particular, the introduction of a 64-slice scanner may be anticipated to have improved the resolution for those cases and it may be speculated that visibility scores could have been improved overall if a 64-slice CT scanner had been used throughout all cases in this study.

This is a retrospective study, and although none of the cats showed any clinical signs nor biochemical evidence of pancreatitis or other pancreatic anomaly, and there was no description of pancreatitis in the history, it is possible that some cats could have had previous pancreatic disease which was not recorded in their history. It is known from postmortem studies that 45% of clinically normal cats may have pancreatitis [1], which suggests that a proportion of our cats that did not show the clinical of biochemical evidence of pancreatitis may have provided histopathological evidence of chronic pancreatitis if histopathology had been available. Nevertheless, this study remains relevant in describing the range of appearance encountered on CT that can be seen in cats clinically unaffected by pancreatic disease.

## 5. Conclusions

CT allows the pancreatic ducts to be identified in most cases, with the left pancreatic duct better visualised than the right and the common ducts. The pancreatic ducts and duodenal papilla are more clearly identified following the IV administration of iodinated contrast medium. Duct measurements did not differ significantly between older cats (aged 10 and over) compared to cats aged 1–9 years.

## Figures and Tables

**Table 1:** Mean ± standard deviation (range) for measurements of the duodenal papilla and of the left, right and common pancreatic ducts, and of Likert visibility scales pre- and post-contrast. Results are presented for each reviewer separately and for all combined data.

	Reviewer 1	Reviewer 2	Both Combined
**Measurements**			
Papilla	2.4 ± 0.6 (1.3–4.0)	3.1 ± 0.7 (1.9–5.3)	2.8 ± 0.7 (1.3–5.3)
Left duct	1.3 ± 0.8 (0.6–7.6)	1.5 ± 0.7 (0.6–4.6)	1.4 ± 0.8 (0.6–7.6)
Right duct	1.2 ± 0.5 (0.5–3.2)	1.1 ± 0.5 (0.3–2.9)	1.1 ± 0.5 (0.3–3.2)
Common duct	1.7 ± 0.8 (0.7–4.6)	1.3 ± 0.5 (0.6–3.7)	1.6 ± 0.8 (0.6–4.6)
**Likert scale**			
Papilla pre	1.2 ± 0.4 (1–3)	1.6 ± 0.8 (1–4)	1.4 ± 0.7 (1–4)
Papilla post	2.5 ± 0.7 (2–5)	2.9 ± 1.2 (1–5)	2.7 ± 1.0 (1–5)
Left pre	1.7 ± 1.0 (1–5)	2.0 ± 1.2 (1–5)	1.9 ± 1.1 (1–5)
Left post	3.4 ± 1.0 (2–5)	3.8 ± 1.3 (1–5)	3.6 ± 1.1 (1–5)
Right pre	1.4 ± 0.8 (1–4)	1.4 ± 1.0 (1–5)	1.4 ± 1.0 (1–5)
Right post	2.8 ± 0.9 (1–5)	2.3 ± 1.5 (1–5)	2.6 ± 1.3 (1–4)
Common pre	1.3 ± 0.7 (1–4)	1.1 ± 0.4 (1–4)	1.2 ± 0.6 (1–4)
Common post	2.9 ± 0.9 (1–5)	2.5 ± 1.4 (1–5)	2.7 ± 1.2 (1–5)

Measurements given in mm to 0.1 mm. All pre-post comparisons for each reviewer, based on Wilcoxon signed rank tests, were significant (all *p* < 0.001).

**Table 2:** Mean ± standard deviation (range) comparing measurements of the pancreatic duct diameter cats aged 1–9 years to cats aged 10+ years. Results are presented both separately for each reviewer and combined.

Duct	Reviewer	1–9 Years	10+ Years	*p*-Value
Left	1	1.1 ± 0.4 (0.6–2.3)	1.4 ± 1.0 (0.6–7.6)	0.125
	2	1.3 ± 0.6 (0.6–2.8)	1.6 ± 0.7 (0.6–4.6)	0.113
	Combined	1.2 ± 0.5 (0.6–2.8)	1.5 ± 0.9 (0.6–7.6)	-
Right	1	1.1 ± 0.4 (0.6–2.6)	1.2 ± 0.6 (0.5–3.2)	0.124
	2	1.1 ± 0.5 (0.3–2.0)	1.1 ± 0.5 (0.3–2.9)	0.984
	Combined	1.1 ± 0.4 (0.3–2.6)	1.2 ± 0.5 (0.3–3.2)	-
Common	1	1.7 ± 0.8 (0.7–4.0)	1.7 ± 0.9 (0.7–4.6)	0.984
	2	1.3 ± 0.7 (0.6–3.7)	1.3 ± 0.4 (0.7–2.2)	0.652
	Combined	1.6 ± 0.8 (0.6–4.0)	1.5 ± 0.7 (0.7–4.6)	-

Measurements given in mm to 0.1 mm. All pre-post comparisons for each reviewer, based on Wilcoxon signed rank tests, were significant (all *p* < 0.001).

**Table 3:** Intraclass correlation coefficients (ICC) for intra and interobserver agreement of measurements and Likert visibility scales.

	Reviewer 1	Reviewer 2	R1 vs. R2
	ICC	ICC	ICC
**Measurements**			
Duodenal papilla diameter	0.66	0.37	0.23
Left pancreatic duct width	0.67	0.93	0.73
Right pancreatic duct width	0.40	0.47	0.76
Common pancreatic duct width	0.24	0.65	0.22
**Likert visibility scales**			
Duodenal papilla (pre)	−0.13	0.40	0.06
Duodenal papilla (post)	0.41	0.62	0.24
Left pancreatic duct (pre)	0.41	0.62	0.59
Left pancreatic duct (post)	0.85	0.88	0.66
Right pancreatic duct (pre)	0.43	0.68	0.59
Right pancreatic duct (post)	0.74	0.81	0.47
Common pancreatic duct (pre)	0.31	−0.04	0.24
Common pancreatic duct (post)	0.67	0.86	0.57

Columns 2 and 3 show intra-observer agreement for reviewers 1 and 2 based on 20 cases assessed 6 weeks apart. Column 4 shows interobserver agreement based on 78 cases.

## Data Availability

The original contributions presented in this study are included in the article. Further inquiries can be directed to the corresponding author.

## Abbreviations

The following abbreviations are used in this manuscript:

HU	Hounsfield unit
CT	Computed tomography
ROI	Region of interest
ALT	Alanine Transaminase
ALKP	Alkaline phosphatase
DGGR	(1,2-o-dilauryl-rac-glycero-3-glutaric acid-(6′-methylresorufin) ester)
fPLI	Serum feline pancreatic lipase immunoreactivity
RCVS	Royal college of veterinary surgeons

## Appendix A

**Table A1:** Final diagnosis of the patient population.

Broad Category	Diagnosis	Number
Neoplasia	Soft tissue sarcoma, including interscapular	12
	Nasal tumour including lymphoma	10
	Pulmonary neoplasia	7
	Mammary tumour	6
	Lymphoma (not nasal)	4
	Thymoma	3
	Osteosarcoma/chondrosarcoma	2
	Iris melanoma	2
	Renal carcinoma	2
	Other—one each of: adrenal tumour, anal sac carcinoma, diffuse carcinoma (thorax), haemangioma, parietal bone mass, retrobulbar, salivary carcinoma, undifferentiated gastric neoplasia, undifferentiated pleural neoplasia	9
Inflammatory/infectious	Respiratory disease including asthma	4
	Pyothorax	2
	Rhinitis (including fungal)	2
	Other—one each of: Aspiration pneumonia, FIP, inflammatory lesion on soft palate, mycobacterial infection from a nasal mass.	4
Autoimmune	Immune mediated polyarthritis	1
Other	One each of: ARDS, Chylothorax, collapsing trachea, Cruciate disease and non-healing wound, Fractured femur, idiopathic chylothorax, pleuroperitoneal hernia, renal disease	8

## Appendix B

**Table A2:** CT protocol at both institutions.

Institution	Scanner	Slices	KVP	Tube Current	Slice Thickness
A	Toshiba Aquillon	16	100	85	0.5 mm
B	GE Revolution Evo	64	120	190	1.25
B	PNMS MX16	16	120	190	1 mm
